# Gratuitous Medical Advice

**Published:** 1877-05

**Authors:** 


### GRATUITOUS MEDICAL ADVICE.

If a hundred individuals, each afflicted with a different ailment, were to come successively into the company of a dozen persons, there is not one of them who would not be told how he could get cured, with the corroborating fact, that the same means had been effectual in the case of "*Mr. Smith, who was a great deal worse off*" than the unfortunate listener, who, if he *tries it*, will only have to add his experience to that of countless thousands who have gone before him, that however efficient it may have proved for others, as for him, it utterly failed of any real good. Or of any hundred newspapers taken up promiscuously, either one of the hundred unfortunates would soon light upon the advertisement of a remedy which suited his case precisely, with the special or implied endorsement of the editor to the same effect, and yet, how has hope been deferred, and the expectant heart sickened and perished in its waiting, all of us understand; and yet, what Mrs. Grundy says, and "I saw it in a newspaper," is gospel to the masses, and to the ignorant amongst the oligarchy of whom there are not a few.

